# *Staphylococcus aureus* Isolated from Ruminants with Mastitis in Northern Greece Dairy Herds: Genetic Relatedness and Phenotypic and Genotypic Characterization

**DOI:** 10.3390/toxins13030176

**Published:** 2021-02-25

**Authors:** Charalampos Kotzamanidis, George Vafeas, Virginia Giantzi, Sofia Anastasiadou, Stavros Mygdalias, Andigoni Malousi, Ekateriniadou Loukia, Sergelidis Daniel, Antonios Zdragas

**Affiliations:** 1 Hellenic Agricultural Organisation-DEMETER, Veterinary Research Institute of Thessaloniki, Campus of Thermi, 57001 Thermi, Greece; vvafeas@vri.gr (G.V.); vgiantzi@vri.gr (V.G.); anastasiadousof@yahoo.gr (S.A.); stavrosmyg@gmail.com (S.M.); ekateriniadou@vri.gr (E.L.); zdragas@vri.gr (A.Z.); 2 Lab of Biological Chemistry, School of Medicine, Aristotle University of Thessaloniki, 54124 Thessaloniki, Greece; andigoni@auth.gr; 3 Laboratory of Hygiene of Foods of Animal Origin—Veterinary Public Health, School of Veterinary Medicine, Aristotle University of Thessaloniki, 54124 Thessaloniki, Greece; dsergkel@vet.auth.gr

**Keywords:** mastitis, *Staphylococcus aureus*, staphylococcal enterotoxin, ruminants, PFGE, *spa* typing, antimicrobial resistance, biofilm, autoaggregation

## Abstract

*Staphylococcus aureus* is the most common mastitis-related pathogen in dairy cattle, goats, and sheep worldwide. However, the population structure and genomic characteristics of mastitis-associated *S. aureus* in small ruminants are limited. Furthermore, the genotypic and phenotypic characteristics involved in the pathogenicity of *S*. *aureus* have been thoroughly defined, yet their association with the severity of mastitis is not fully established. Here, we performed genotyping by pulsed-field gel electrophoresis (PFGE) and *spa* analyses to assess the genetic diversity and relatedness of 162 *S. aureus* strains recovered from clinical mastitis (CM) and subclinical mastitis (SCM) cases from goats, sheep, and bovines. PFGE analysis revealed 108 distinguishable pulsotypes and 3 main clusters that comprised isolates from the three host species, while according to *spa* typing, 32 different *spa* types were identified. Genotypic analysis revealed a spreading of genetically related or indistinguishable *S. aureus* strains among ovine, caprine, and bovine farms of distant geographical regions. In total, 28 different staphylococcal enterotoxin (SE) gene profiles were observed, revealing a diverse range of SE genes among isolates. By evaluating the antimicrobial resistance, we found low phenotypic antimicrobial resistance among all ruminant isolates. We also performed multiple correspondence analysis, which indicated that the presence of the *sec* gene, biofilm production, and high autoaggregation ability are associated with CM cases.

## 1. Introduction

Mammary infection of ruminants leading to subclinical and clinical mastitis is associated with severe economic losses due to the reduction in milk yield and milk quality and the cost of treatment [1] and has also an impact on animal welfare [2]. *Staphylococcus aureus* is the most common mastitis-related pathogen in dairy cattle, goats, and sheep [3,4], with infected animals frequently shedding the bacterium into their milk [5]. In humans, epidemiological studies have identified *S. aureus* as the most common agent involved in food poisoning upon ingestion of foods containing pre-formed staphylococcal enterotoxins (SEs) [6]. Furthermore, methicillin-resistant *S. aureus* (MRSA) constitutes a serious public health concern, and it has been proved that infections in the community and subsequent foodborne outbreaks can also be caused by livestock-associated (LA) MRSA, due to the handling and consumption of contaminated food of animal origin [7].

Intramammary infection in animals is caused by *S. aureus* strains with the capacity to produce a variety of virulence factors such as antigens, toxins, and various resistance proteins [8]. Staphylococcal enterotoxins are considered to play an important role in the development of mastitis, since *S. aureus* isolates from cases of mastitis had a higher prevalence of enterotoxin genes than isolates from milk of cows without mastitis [9], and in addition, SE genes were found to be related with the severity of bovine mastitis [10]. The ability of *S. aureus* to form biofilm is also considered a crucial virulence trait [11], while the *S. aureus* property of autoaggregation is often associated with pathogenicity [12].

Although the population structure and genomic characteristics of *S. aureus* from bovines have been reported, the number of systematic studies and analogous data for *S. aureus* isolated from small ruminants, and in particular goats, is very limited [11,13]. Greece is one of the European countries with a large population of small ruminants. Numerous studies have identified that *S. aureus* is one of the most commonly found pathogens in bovine, caprine, and ovine raw milk, while the bacterium has been detected in 40%, 31–80%, and 24–63% of the examined bulk tank milk from Greek dairy bovine, goat, and sheep farms, respectively [14,15,16].

Previous studies have shown that *S. aureus* from goats and sheep are closely related and distinct to bovine ones and that there is a different clonal composition between small ruminants and cows. In these studies, different genotyping methods, including *spa* sequence typing, ribosomal spacer PCR (RS-PCR) virulence gene profiling, and multilocus sequence typing (MLST) [13,17,18], have been used for the characterization of *S. aureus* strains, enabling to evaluate their genetic relatedness. In contrast, other studies based on pulsed-field gel electrophoresis (PFGE) analysis have shown that closely related mastitis-associated genotypes of *S. aureus* are capable of colonizing and infecting different host species such as sheep, goats, and bovines [19,20,21]. The discriminatory power of PFGE is considered relatively higher compared to other typing methods such as MLST and *spa* typing [22]. However, a general lack of information exists regarding large-scale studies based on PFGE for the genotyping and the subsequent investigation of *S. aureus* population structure in small ruminants.

Since there is little information about and contradictory results on the genomic population structure of mastitis-associated *S. aureus* recovered from sheep, goats, and bovines, the ecology and transmission of this bacterium in the farm environment remains unclear and studies on the characterization of clinical mastitis (CM) as well as subclinical mastitis (SCM)-associated isolates from small ruminants are needed. In previous works we investigated the prevalence of *S. aureus* and MRSA in cattle, sheep, and goat farms as well as in dairy industries in Greece, and we also accessed their genetic diversity [16,23]. In this study, to obtain more insights into the transmission routes and infection sources of this important pathogen in the farm environment, we presented molecular typing analyses, antimicrobial susceptibility testing, and cell surface traits to characterize and compare bovine, ovine, and caprine *S. aureus* isolates from CM and SCM cases. The objectives of the present study were (i) to describe by PFGE and *spa*-typing analysis the genetic diversity of *S. aureus* isolated from cases of ovine, caprine, and bovine mastitis in order to evaluate the population structure and genetic relatedness of *S. aureus* recovered from different ruminant species and (ii) to investigate whether specific genomic and phenotypic characteristics of the pathogen are associated with CM or SCM in ruminants.

## 2. Results

### 2.1. Distribution of Virulence and Resistance Genes

All the 162 ruminant isolates tested positive for *coa* and the species-specific *nuc* genes, confirming the phenotypic identification of the selected *S. aureus* isolates. However, none of the isolates tested positive for the presence of either of the methicillin resistance genes, *mec*A or *mec*C. Concerning *S*. *aureus* toxins, one or more of the 10 types were detected in 109 (67.3%, 109/162) of the analyzed *S*. *aureus* isolates (Table 1).

The *sec* (32.7%, 53/162), *seb* (27.1%, 44/162), sei (17.9%, 29/162), and *sej* (13.0%, 21/162) genes were the most common genes detected among all the isolates, followed by *sea* (6.2%, 10/162), *sed* (4.3%, 7/162), *seg* (3.7%, 6/162), and *seh* (1.9%, 3/162), while none of the isolates carried the *see* gene. The *tsst* gene was only identified in two *S. aureus* isolates (1.2%, 2/162), one ovine and one bovine, while none of the isolates tested positive for the *luk*F-PV gene encoding the Panton–Valentine leukocidin (PVL) toxin. One or more enterotoxin genes were detected simultaneously in 38.9% (63/162) of all 162 isolates, and in total, 28 different virulence gene profiles were observed. Among them, the most frequent toxin–gene combinations were *seb*, *sec* (9 isolates) and *seb*, *sei* (8 isolates). The largest diversity, 15 toxin gene profiles, was found among ovine *S. aureus*, followed by caprine and bovine isolates, with 12 and 7 profiles, respectively.

### 2.2. PFGE Genotyping

**Figure 1:**
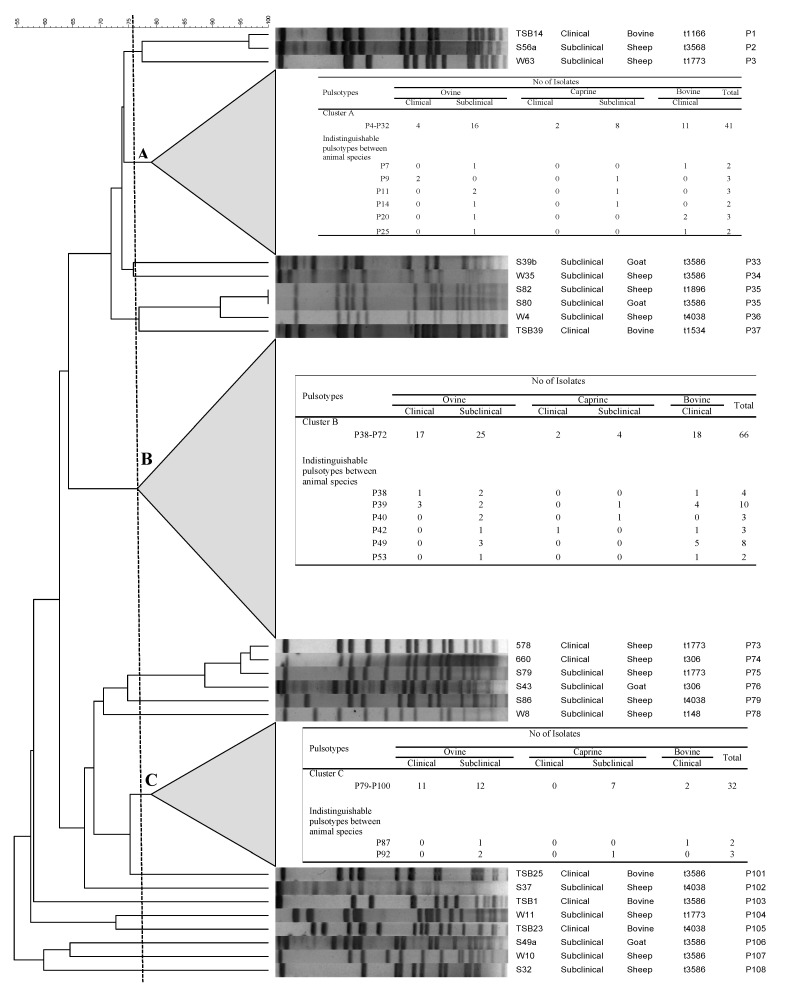
Dendrogram of *sma*I pulsed-field gel electrophoresis (PFGE) macrorestriction patterns of *S. aureus* isolated from cases of ovine, caprine, and bovine mastitis. The dendrogram is based on analysis by the unweighted pair group with the arithmetic averages clustering method. Clusters A, B, and C were defined at a similarity level of 75%.

All the 162 *S*. *aureus* isolates were typeable by PFGE following digestion by restriction enzyme smaI, revealing 108 distinguishable pulsotypes (59 among 97 ovine isolates, 25 among 29 caprine isolates, 24 among 36 bovine isolates) (Figure 1 and Figure S1).

The Simpson index of discrimination was 0.990. At a similarity level of 75% or above, the majority of *S. aureus* isolates (83.3%, 135/162) were assigned into three clusters (A, B, and C), which shows complete host diversity, indicating the absence of association with host species for these clusters. In more detail, cluster A consisted of ovine (20), caprine (10), and bovine (11) isolates obtained from CM or SCM cases of the three host species. Looking at B, 39 ovine, 6 caprine, and 18 bovine clinical- or subclinical-mastitis-associated isolates belonged to this cluster. Finally, cluster C almost consisted of ovine and caprine isolates, containing 23 ovine, 7 caprine, and 2 bovine isolates from both CM and SCM cases.

Interestingly, indistinguishable pulsotypes shared by ovine, caprine, and bovine isolates were identified: pulsotypes P7, P20, P25, P38, P42, P49, P53, and P87 were shared by ovine and bovine isolates; pulsotypes P9, P11, P14, P35, P40, and P92 were shared by ovine and caprine isolates; while P39 and P42 were common to all ruminant species. Furthermore, looking at indistinguishable pulsotypes, we could identify common pulsotypes among isolates obtained from CM and SCM cases (P7, P9, P20, P25, P38, P39, P42, P49, P53, P87).

### 2.3. Identification and Distribution of spa Types

**Figure 2:**
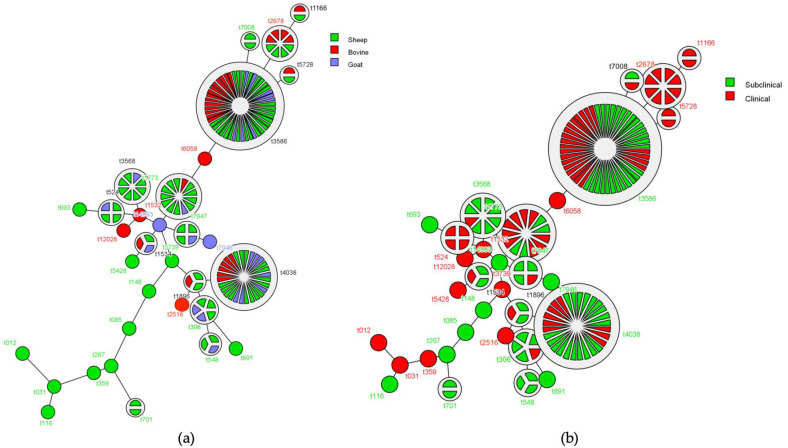
Minimum spanning trees based on *spa*-typing results of ovine, caprine, and bovine *S*. *aureus* mastitis isolates, depending on the host animal (**a**) and the severity of mastitis (**b**). Each *spa* type is depicted by a single node. The size of the node is proportional to the number of isolates within the *spa* type, while colored sections represent the host species of the isolates or the severity of mastitis. The distance between the nodes represents the genetic divergence.

To access the clonal profile and diversity of the *S*. *aureus* population, *spa* typing and PFGE were performed. According to *spa* typing, 32 different spa types were identified and the majority of them (17/32) were represented from single isolates, revealing the high diversity of the *S*. *aureus* population (Table 2). Among ovine isolates, a diverse range of *spa* types was detected (27 *spa* types), while the caprine- and bovine-associated isolates belonged to 10 and 12 different *spa* types, respectively. The most prevalent *spa* types were t3586 (n = 48; 30.0%), t4038 (n = 28; 17.3%), and t1773 (n = 13; 8.0%), each of them consisting of isolates from all host species, comprising the majority (53 ovine, 15 caprine, 21 bovine; 55.0%) of all isolates. Two minimum spanning trees based on *spa*-typing results of the *S*. *aureus* population, depending on the host animal (Figure 2a) and the severity of mastitis (Figure 2b), were constructed. These graphs showed the *spa* type frequencies and the genetic distance between them. The graph in Figure 2a illustrates a marked diversity among ovine, caprine, and bovine isolates, while Figure 2b shows that specific *spa* types are exclusively associated with CM (t1166, t2678, t5728, and t524) or SCM (t548) in different animal species.

### 2.4. Phenotypic Antimicrobial Resistance

According to antimicrobial susceptibility testing, resistance of *S. aureus* isolates to 4 of the 20 antimicrobial agents tested was observed (Table 3). Among all isolates, 43.2% (70/162) were found to be resistant to at least one antimicrobial agent, with the highest resistance frequency being observed to penicillin (16.7%, 27/162) and tetracycline (14.2%, 23/162), followed by streptomycin (10.5%, 17/162) and erythromycin (1.9%, 3/162). In addition, *S. aureus* isolates from bovines exhibited the highest resistance frequency to penicillin (25.0%, 9/36) and tetracycline (25.0%, 9/36) compared with sheep and goat isolates. Regarding multiresistance, 8.6% (14/162) of the isolates were resistant to more than one antibiotic. Overall, seven antimicrobial resistance profiles (ARPs) were defined, while two isolates (1.2%) were characterized as multidrug resistant (MDR), exhibiting resistance to three different classes of antimicrobials.

### 2.5. Biofilm Formation and Cell Surface Traits

The biofilm formation assay differentiated *S. aureus* isolates into strong, moderate, and weak (Table 3 and Figure S2). The majority (60.4%, 98/162) of isolates were characterized as strong biofilm producers, followed by isolates with moderate (34%, 55/162) and weak (5.6%, 9/162) biofilm formation capacity. With respect to autoaggregation, the majority (61.7%, 100/162) of the *S. aureus* isolates belonged to the group with low ability. Biofilm formation strength (chi-square = 11.603; *p* = 0.003; significant association at *p* < 0.01) and autoaggregation ability (chi-square = 59.043; *p* < 0.0001; significant association at *p* < 0.01) were significantly associated with the severity of mastitis, while all isolates exhibited low cell surface hydrophobicity.

### 2.6. Associations between SE Genes, Genotypic Traits, and Mastitis

**Figure 3:**
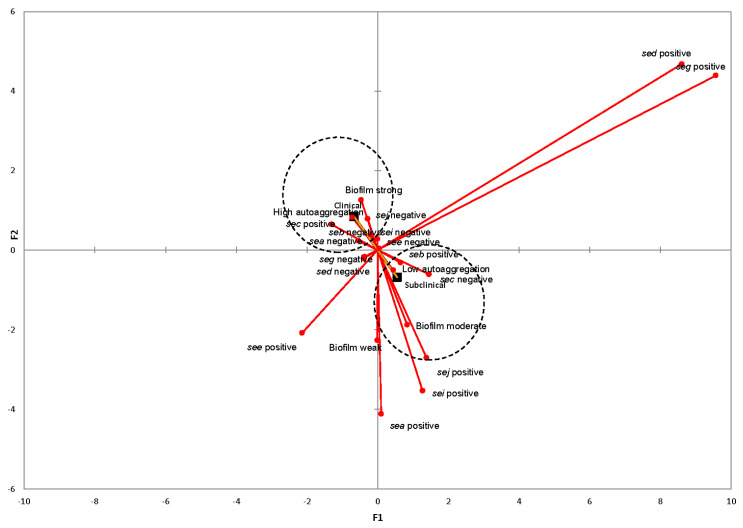
Multiple correspondence analysis (MCA) for the correlation between biofilm formation strength, autoaggregation ability, the presence of virulence genes, and the severity of mastitis of *S. aureus* isolated from cases of ovine, caprine, and bovine clinical and subclinical mastitis. The first two factorial axes display 32.3% and 22.55% of the total variability, respectively. Vectors with blocks (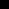) represent clinical and subclinical mastitis cases.

The first two factorial axes produced by multiple correspondence analysis (MCA) were used to compose the factorial plane 1 × 2. These two axes represent 32.3% and 22.55%, respectively, of the total inertia, explaining together 54.85% of the total variability (Figure 3). It can be observed that the second factorial axis mainly differentiates clinical from subclinical isolates. Correspondence analysis investigating the interaction between biofilm formation, autoaggregation ability, virulence genes, and the severity of mastitis showed that the presence of the *sec* gene as well as the strong-biofilm-producing and high-autoaggregating *S. aureus* was associated with CM. On the other hand, moderate-biofilm-forming and low-autoaggregating *S. aureus* isolates were related to SCM, while the *sed* and *seg* genes were not associated with either CM or SCM.

The results were further tested with a series of chi-square independence tests, which confirmed that the enterotoxin genes (chi-square = 26.753; *p* = 0.0001; significant association at *p* < 0.01) were significantly associated with the severity of mastitis.

## 3. Discussion

Considering that *S. aureus* is the major cause of mastitis, one of the costliest dairy farm diseases worldwide, and that little information is available regarding the molecular epidemiology of this pathogen in small ruminants, the main objective of this study was to elucidate the population structure and genetic relatedness of *S. aureus* recovered from sheep, goats, and bovines. To this end, we performed genotyping based on PFGE analysis. In consistence with previous studies, genetic characterization of the isolates by PFGE revealed that ruminants are exposed to a broad range of genetically diverse strains in the farm environment. Genomic variability among the 162 *S. aureus* isolates was demonstrated by the large number (108) of distinct PFGE patterns, as well as the high value of Simpson’s index of diversity (D = 0.990).

The majority of the ovine, caprine, and bovine isolates (83.3%, 135/162) were grouped together into three main clusters (A, B, and C). Interestingly, all host species in clusters A and B were present in significant proportion, showing host diversity and the absence of association with host species for these clusters. On the other hand, the fact that cluster C comprised almost exclusively ovine and caprine isolates indicates that *S. aureus* from small ruminants may show a closer genetic relationship and also may form a population distinct from the bovine isolates. The main clusters that have been revealed can support the hypothesis that during a period time of five years, in the region of northern Greece, three main clones defined by PFGE analysis were shared by ovine, caprine, and bovine mastitis-associated *S. aureus* and started to spread among farms of distant geographical regions. This finding is in agreement, in part, with a previous study that revealed in Greece the existence of similar clones of *S. aureus* isolated from SCM cases of dairy goats among distantly located herds [11]. Studying the genetic relationships between isolates is important for understanding the transmission and spread of a pathogen. However, genetic information obtained is influenced by the choice of method, different sample sizes, type of farming systems, and geographical conditions [18]. In this aspect, previous studies based on MLST, RS-PCR, and s*p*a typing [13,17,18] have demonstrated the genetic diversity of *S. aureus* from ruminants as well as the fact that ovine and caprine isolates are similar and distinct from cattle isolates. Our work, based on PFGE analysis, clearly demonstrates not only the genetic diversity of the *S. aureus* population but the existence of PFGE clones that comprised isolates with considerable genetic similarity from all host species, indicating that *S. aureus* isolates from sheep, goats, and bovines do not represent separate genetic populations. These findings are in line with previous studies that were also based on PFGE [20,24,25]. Thus, taking into consideration its high discriminatory power, we conclude that the PFGE method is capable of revealing the population structure of mastitis-associated *S. aureus* recovered from different ruminant species in a specific geographical region. Furthermore, a key finding of the current study was the detection of indistinguishable *S. aureus* pulsotypes from ovine, caprine, and bovine hosts, indicating the existence of specific pathogen strains with the capacity to cause disease in different host species. In addition, the existence of these indistinguishable *S. aureus* isolates is suggestive of bacterial transmission between different animal species/herds.

To better elucidate the population structure of mastitis-associated *S. aureus* from ruminants, we performed *spa* typing, which also revealed a remarkable genetic diversity among isolates. We found three *spa* types (t3586, t4038, and t1773) that contain isolates from all host species, comprising the majority of all isolates. We also found specific *spa* types that are exclusively associated with CM (t1166, t2678, t5728, and t524) or SCM (t548) in different animal species. In agreement with PFGE, the above findings also support the idea that *S. aureus* isolates from sheep, goats, and bovines do not represent separate genetic populations. Regarding the most prevalent *spa* types in our study, t1773 was also reported by Merz [17] and Romano [13] as the most prevalent *spa* type in caprine isolates in Switzerland and northern Italy, respectively, while t3586 and t4038 types were identified among MRSA isolates in Greece, from sheep and goats, respectively [15,16].

Overall, our findings support the hypothesis that *S. aureus* from ruminants is a clonal microorganism that spreads among different animal species, even among farms of distant geographical regions, implying that adaptive evolution of *S. aureus* has led to host dissemination. Close genetic relation as well as transmission of indistinguishable isolates among ovine and caprine isolates from distant herds are most likely associated with the low-input systems of goat and sheep production, which are the dominant systems practiced in Greece [11]. However, the underlying reasons for the spread of genetically related or indistinguishable *S. aureus* isolates among ovine, caprine, and bovine farms of distant geographical regions in northern Greece remain unclear, and further studies from broad geographical origins are needed to elucidate transmissibility between different host species, ensuring that this finding can be extended to the general *S. aureus* population.

Another focus of this study was the characterization of *S. aureus* isolates and the identification of specific genomic and phenotypic characteristics of the pathogen that could be associated with the outcome of mastitis in ruminants. To this end, we evaluated antimicrobial resistance; microbial cell surface traits such as the aggregation ability, hydrophobicity, and biofilm formation capacity; as well as virulence and resistance genes contributing to *S. aureus* pathogenicity.

Overall, by evaluating antimicrobial resistance, we found a low phenotypic antimicrobial resistance among all ruminant isolates. Since the cell surface hydrophobicity of *S*. *aureus* has been suggested to contribute as an adaptive reaction against antimicrobial agents [26], the low hydrophobicity we observed among all isolates is in accordance with the low antimicrobial resistance. A relatively higher resistance frequency was observed to penicillin (16.7%, 27/162) and tetracycline (14.2%, 23/162), consistent with previous studies in Greece [14] and in Europe [22,27] that noted penicillin or tetracycline as the primary resistance phenotypes. The low frequency of resistance to antimicrobials such as penicillin and tetracycline, which they usually used in veterinary medicine to cure mastitis, is strong evidence of the low pressure of antimicrobials in small ruminants in Greece [14]. A relatively higher resistance frequency to penicillin and tetracycline was observed among bovine isolates. This is indicative of a higher selective pressure due to the more frequent use of antimicrobials in bovines compared with low-input traditional goat and sheep herds, in Greece.

Regarding the virulence and resistance genes contributing to *S. aureus* pathogenicity, none of the isolates tested positive for the *luk*F-PV gene encoding the Panton–Valentine leukocidin (PVL) toxin and for the methicillin resistance genes, *mec*A or *mec*C; the latter confirms the evidence of the low isolation frequency of MRSA among isolates from goat, sheep, and bovine raw milk in Greece, as previously described [11,14,15,16]. We found a high number of different virulence gene profiles, revealing a diverse range of SE genes among isolates. The most frequent SE genes among the *S*. *aureus* isolates were *sec* and *seb*, while all isolates tested negative for *see*. Similar studies have also reported a high prevalence of the *sec* and *seb* genes among *S*. *aureus* isolated from small ruminants and bovines with mastitis [8,15], while the absence of the enterotoxin gene *see* in our isolates is in accordance with previous results [10].

Numerous studies were accomplished in order to associate phenotypic and genotypic characteristics of *S*. *aureus* with the clinical or subclinical manifestation of mastitis in small ruminants and bovines [28,29,30,31,32]. In this aspect, we performed multiple correspondence analysis, revealing that the presence of the *sec* gene is associated with CM. These results corroborate those of previous studies that reported that *sec* is the most frequently isolated gene in animals suffering from mastitis [33,34] and is recognized as an important virulence factor in bovine mastitis [31]. In addition, Fang et al. [33], using a mouse model, demonstrated that the enterotoxin SEC can directly cause inflammation, proinflammatory cytokine production, and tissue damage in mammary glands, suggesting that it might play an important role in the development of mastitis associated with *S. aureus* infection. Interestingly, correspondence analysis showed that the detection of the enterotoxin *seg* gene does not correlate with either CM or SCM, confirming the results of a previous study that stated that the *seg* gene is associated with a decreased likelihood of bacteria causing intramammary infections during lactation in dairy cows [35]. Regarding *S. aureus* cell surface properties, multiple correspondence analysis demonstrated that strong-biofilm-producing and high-autoaggregating *S. aureus* isolates were associated with CM, while moderate-biofilm-forming and low-autoaggregating isolates were related to SCM. Several studies have suggested that biofilm formation and autoaggregation are associated with epithelial adhesion and resistance to antimicrobial agents, enabling *S*. *aureus* to resist host immune defense mechanisms, and therefore are considered as important factors in the pathogenesis of intramammary infections [12,36,37].

In summary, this study revealed that sheep, goats, and bovines are exposed to a broad range of genetically diverse strains of mastitis-associated *S*. *aureus* that possess variable enterotoxin genes. We also provided evidence that *S. aureus* from ruminants is a clonal microorganism that spreads among different animal species and that pathogen isolates from sheep, goats, and bovines do not represent separate genetic populations. Furthermore, we showed that specific pathogen strains have the capacity to cause mastitis in different host species, even among farms of distant geographical regions. We also demonstrated that the outcome of mastitis in ruminants is associated with specific genotypic and phenotypic traits of *S*. *aureus*. However, the pathogenesis of *S. aureus* mastitis is complex, depending on host–pathogen interactions, which are determined by numerous factors such as host health, expression of virulence genes, and host immune system. Thus, it is likely that specific phenotypic traits such as biofilm capacity and aggregation ability as well as the presence of virulent genes alone cannot define the outcome of interactions with the host in vivo and the outcome of mastitis [28,30]. Therefore, further data are needed to understand the epidemiology as well as the bacterial factors that are associated with the severity of mastitis in ruminants. This work highlights the importance of understanding the population structure, transmission, virulence characteristics, and pathogenicity of *S*. *aureus* from ruminant mastitis in order to develop strategies for reducing the spread of the pathogen among herds in a specific geographical region.

## 4. Materials and Methods

### 4.1. Bacterial Strains

A total of 162 *S. aureus* isolates obtained from cases of ovine (n = 97), caprine (n = 29), and bovine (n = 36) CM or SCM were analyzed in this study. *S. aureus* isolates were selected from the strain collection of staphylococci maintained at our laboratory that comprised isolates collected at different time points over a period of approximately five years from farms located in northern Greece. *S. aureus* from SCM cases (25 caprine and 63 ovine) were obtained from bulk tank milk (BTM) samples from SCM-positive farms, whereas CM isolates (4 caprine, 34 ovine, and 36 bovine) were obtained after cultivation on agar plates of individual milk samples from animals suffering from CM. For BTM sampling, the criteria for selecting ovine and caprine farms positive for SCM were the presence of animals without any CM signs that produced milk with a somatic (body-derived; mainly leucocytes) cell count (SCC) of >10^6^/mL measured by means of an automatic high-throughput analyzer (Fossomatic FC; Foss, Hilleroed, Denmark)—a value that indicates the manifestation of SCM [38]. For individual milk samples, the selection of animals with CM was based on the diagnosis of clinical signs of inflammation on the mammary gland (swelling, pain), macroscopic abnormalities in the milk (pus, lumps, and blood streaks), increased somatic cell counts, a positive California mastitis test, and decreased milk yield [38].

Isolation of staphylococci from milk samples was carried out on Baird–Parker agar supplemented with Egg-Yolk Tellurite (LAB M, Lancashire, UK) after incubation for 48 h at 37 °C, as previously described [11]. From plates displaying bacterial growth, up to four well-isolated black colonies, with or without an opaque halo (presumptive *Staphylococcus* spp.), were randomly selected and sub-cultured onto Tryptone Soya Yeast Extract agar (LAB M, Lancashire, UK) for 24 h at 37 °C for purification and further characterization. Identification of *S. aureus* strains was based upon biochemical assays (Gram staining, catalase reaction, hemolysis, mannitol fermentation, tube coagulase test, and V-P reaction), followed by PCR tests for the detection of *coa* and the species-specific *nuc* genes. Overall, only one randomly chosen isolate per farm, containing sheep or goats or cows only, was considered for this study.

### 4.2. DNA Extraction and Species Confirmation

All phenotypically confirmed *S. aureus* strains were subjected to further molecular characterization. A DNA purification protocol for Gram-positive bacteria (Pure Link Genomic DNA kit; Invitrogen, Carlsbad, CA, USA) was used for genomic DNA extraction. The PCR conditions previously described by Zdragas et al. [14] were used for the detection of *coa* [39] and the species-specific *nuc* [40] genes in order to confirm the identification of all presumptive *S. aureus* isolates.

### 4.3. Detection of Virulence and Resistance Genes

Multiplex PCRs were used for the detection of virulence and resistance genes contributing to *S. aureus* pathogenicity. Two sets of primers (*sea*, *seb*, *sec*, *seh*, and *sej*; *sed*, *see*, *seg*, *sei*, and *tsst*) were used to detect the genes of 10 types of SEs. PCR amplification and amplicon analysis by electrophoresis were performed, as described by Lovseth et al. [41]. The methicillin resistance genes (*mec*A, *mec*C) and the *luk*F-PV gene encoding the Panton–Valentine leukocidin (PVL) toxin were detected by multiplex PCR using the primers and conditions described by Stegger et al. [42].

### 4.4. PFGE

PFGE analysis of *S. aureus* isolates was performed according to the PulseNet protocol [43] with the *sma*I enzyme (New England Biolabs, Beverly, MA, USA) by using a CHEF-DR III apparatus (Bio-Rad Laboratories Inc., Hercules, CA, USA) for the separation of DNA fragments. *Xba*I-digested DNA from *Salmonella enterica* serotype Braenderup H9812 was used as a reference size standard, while PFGE patterns were digitally analyzed using the FPQuest (Bio-Rad Laboratories Pty Ltd. Hercules, CA, USA) software package. PFGE profiles were compared using the Dice correlation coefficient, with a maximum position tolerance of 1.5% and optimization of 1.5%. Similarity, clustering analysis was performed using the unweighted pair group method using averages (UPGMA), and a dendrogram was generated. Two PFGE profiles were classified as indistinguishable if the DNA fragment patterns matched each other completely, while clusters were selected using a cutoff at the 75% level of genetic similarity. The discriminatory power of PFGE analysis was determined using Simpson’s index of discrimination (D), as previously described [44]. Values for D ranged between 0 and 1, with a value of 1 indicating the most discriminatory method.

### 4.5. Spa Typing

*spa* typing was performed according to Aires-de-Sousa et al. [24]. The sequence data of the polymorphic regions of the *spa* gene were analyzed using the *spa*-typing plugin in BioNumerics v.8.0 software (Applied Maths, Sint-Martens-Latem, Belgium; under a temporary evaluation license; receiving permission to publish), which connects to the SeqNet/Ridom Spa Server (https://www.spaserver.ridom.de/ accessed on 25 February 2021). A minimum spanning tree (MST) was also generated in BioNumerics using the *spa*-clustering method of the *spa*-typing plugin.

### 4.6. Biofilm Formation and Cell Surface Traits

#### 4.6.1. Biofilm Formation

The biofilm formation capacity of staphylococcal strains was determined using a semi-quantitative, microtiter-plate (MTP) adherence assay according to the protocol described by Wang et al. [45], which measures the optical density (OD) of adherent biofilms stained with 0.3% (w/v) crystal violet at 570 nm. The cutoff optical density (ODc) was defined as the mean OD value of the negative control (plain broth medium). Depending on the resulting OD readings, *S. aureus* strains were classified according to Borges et al. (2012) [46] as no biofilm producers (OD < ODc), weak biofilm producers (ODc < OD ≤ 2 × ODc), moderate biofilm producers (2 × ODc < OD ≤ 4 × OD_C_), or strong biofilm producers (4 × ODc < OD).

#### 4.6.2. Autoaggregation Assay

Autoaggregation assay was performed, as described previously [47], with slight modifications. Briefly, 4 mL of phosphate-buffered saline (PBS) containing 10^8^ CFU/mL of bacterial cells was resuspended by vortexing for 10 s and incubated for 5 h at room temperature. At 1 h intervals, 0.1 mL of the upper suspension was carefully removed and transferred to another tube containing 3.9 mL of PBS, and the A600 was measured. The experiment was repeated three times, and the autoaggregation percentage was expressed as a function of time until it was constant, using the following formula: Autoaggregation (%) = 1 − (At/A0) × 100, where At represents the absorbance at time t = 1, 2, 3, 4, or 5 h and A0 is the absorbance at t = 0. The degree of bacterial aggregation was classified as low (0–69%) or high (70–100%) [48].

#### 4.6.3. Cell Surface Hydrophobicity

Bacterial cell surface hydrophobicity was determined by measuring microbial adhesion to hydrocarbons (MATH), as previously described [49], by using different organic solvents such as xylene and n-dodecane. Cells in the stationary phase were washed twice in PBS and finally re-suspended in 3 mL of 0.1 M KNO_3_ containing about 10^8^ CFU/mL of bacteria, and absorbance was measured at 600 nm (A0). One milliliter of xylene was then added to the cell suspension to form a two-phase system. After 10 min pre-incubation at room temperature, the two-phase system was mixed by vortexing for 2 min. Then, the water and organic solvent phases were allowed to separate by incubation for 20 min at room temperature. The aqueous phase was carefully removed, and its absorbance (A1) was measured at 600 nm, while KNO_3_ was used as a control. The experiment was repeated three times, and the percentage of cell surface hydrophobicity (H%) was calculated using the following formula: H(%) = (1 − A1/A0) × 100. The degree of bacterial hydrophobicity was classified as low (0–29%), medium (30–59%), or high (60–100%) [50].

### 4.7. Antimicrobial Susceptibility Testing

*S. aureus* isolates were tested for their susceptibility by the standard disk diffusion method on Mueller–Hinton agar (Merck, Darmstadt, Germany) using commercially available discs (Oxoid, Basingstoke, UK) according to the Clinical and Laboratory Standard Institute (CLSI) guidelines [51]. The antimicrobial agents used were penicillin, cefoxitin, ceftazidime, amikacin, gentamicin, kanamycin, streptomycin, chloramphenicol, clindamycin, erythromycin, ciprofloxacin, tetracycline, trimethoprim/sulphamethoxazole, rifampicin, cephalothin, cefotaxime, ceftriaxone, vancomycin, ofloxacine, and tombramycin. Multidrug resistance was defined, as previously proposed [52]. *S. aureus* ATTC 25923 was used as the control strain.

### 4.8. Statistical Analysis

All experiments were carried out in triplicate. All statistical analyses were performed using XLSTAT software, version 2014.5.03 (Addinsoft, New York, NY, USA). Comparisons of proportions were evaluated using the chi-square test or Fisher’s exact test, as appropriate. To understand the relationship between specific genotypic (enterotoxin genes) and phenotypic (biofilm formation strength, autoaggregation ability) characteristics of isolates and the severity of mastitis, multiple correspondence analysis (MCA) was performed. As the MCA method is not designed for hypothesis testing, interrelation of the analyzed variables was also examined by the chi-square test to further support the results of MCA. *p* < 0.05 was considered statistically significant [53].

## Figures and Tables

**Table 1:** Detection of toxin genes in *Staphylococcus* *aureus* strains isolated from cases of ovine, caprine, and bovine mastitis.

	No. of Isolates (%)					
	Ovine (n = 97)		Caprine (n = 29)		Bovine (n = 36)	Total (n = 162)
	Clinical (n = 34)	Subclinical (n = 63)	Clinical (n = 4)	Subclinical (n = 25)	Clinical	
Toxin genes						
*sea*	0 (0)	9 (14.3)	0 (0)	1 (4.0)	0 (0)	10 (6.2)
*seb*	5 (14.7)	19 (30.2)	1 (25.0)	7 (28.0)	12 (33.3)	44 (27.1)
*sec*	11 (32.3)	18 (28.5)	0 (0)	8 (32.0)	16 (44.4)	53 (32.7)
*sed*	0 (0)	4 (6.3)	0 (0)	8 (0)	1 (2.8)	7 (4.3)
*see*	0 (0)	0 (0)	0 (0)	0 (0)	0 (0)	0 (0)
*seh*	0 (0)	3 (4.8)	0 (0)	0 (0)	0 (0)	3 (1.9)
*seg*	1 (2.9)	3 (4.8)	0 (0)	2 (8.0)	0 (0)	6 (3.7)
*sei*	4 (11.8)	18 (28.5)	0 (0)	7 (28.0)	0 (0)	29 (17.9)
*sej*	1 (2.9)	8 (12.7)	1 (25.0)	3 (12.0)	8 (22.2)	21 (13.0)
*pvl*	0 (0)	0 (0)	0 (0)	0 (0)	0 (0)	0 (0)
*tsst*	1 (2.9)	0 (0)	0 (0)	0 (0)	1 (2.8)	2 (1.2)
Toxin gene patterns						
*sea*	0 (0)	2 (3.2)	0 (0)	0 (0)	0 (0)	2 (1.2)
*seb*	2 (5.9)	5 (7.9)	1 (25.0)	1 (4.0)	2 (5.5)	11 (6.7)
*sec*	11 (32.3)	10 (15.9)	0 (0)	0 (0)	10 (27.8)	34 (21.0)
*sed*	0 (0)	1 (1.6)	0 (0)	0 (0)	0 (0)	1 (0.6)
*seh*	0 (0)	1 (1.6)	0 (0)	0 (0)	0 (0)	1 (0.6)
*sei*	0 (0)	6 (9.5)	0 (0)	0 (0)	0 (0)	6 (3.7)
*sej*	0 (0)	0 (0)	1 (25.0)	1 (4.0)	5 (13.9)	7 (4.3)
*tsst*	1 (2.9)	0 (0)	0 (0)	0 (0)	0 (0)	1 (0.6)
*sea*, *seb*	0 (0)	1 (1.6)	0 (0)	0 (0)	0 (0)	1 (0.6)
*sea*, *sei*	0 (0)	0 (0)	0 (0)	1 (4.0)	0 (0)	1 (0.6)
*sea*, *sej*	0 (0)	1 (1.6)	0 (0)	0 (0)	0 (0)	1 (0.6)
*seb*, *sec*	0 (0)	2 (3.2)	0 (0)	1 (4.0)	6 (16.7)	9 (5.6)
*seb, sei*	2 (5.9)	4 (6.3)	0 (0)	2 (8.0)	0 (0)	8 (4.9)
*seb*, *sej*	0 (0)	0 (0)	0 (0)	0 (0)	3 (8.3)	3 (1.9)
*sec*, *sei*	0 (0)	0 (0)	0 (0)	1 (4.0)	0 (0)	1 (0.6)
*sec, sej*	0 (0)	0 (0)	0 (0)	1 (4.0)	0 (0)	1 (0.6)
*sed*, *seg*	0 (0)	0 (0)	0 (0)	1 (4.0)	0 (0)	1 (0.6)
*sei*, *sej*	2 (5.9)	3 (4.8)	0 (0)	0 (0)	0 (0)	4 (2.5)
*sea*, *sec*, *seh*	0 (0)	2 (3.2)	0 (0)	0 (0)	0 (0)	2 (1.2)
*sea*, *sei*, *sej*	0 (0)	0 (0)	0 (0)	0 (0)	1 (2.8)	1 (0.6)
*seb*, *sec*, *sei*	0 (0)	1 (1.6)	0 (0)	2 (8.0)	0 (0)	3 (1.9)
*seb*, *sed*, *seg*	0 (0)	2 (3.2)	0 (0)	1 (4.0)	0 (0)	3 (1.9)
*seb*, *sed*, *tsst*	0 (0)	0 (0)	0 (0)	0 (0)	1 (2.8)	1 (0.6)
*seb*, *seg*, *sei*	1 (2.9)	0 (0)	0 (0)	0 (0)	0 (0)	1 (0.6)
*sed*, *seg*, *sej*	0 (0)	1 (1.6)	0 (0)	0 (0)	0 (0)	1 (0.6)
*sea*, *seb*, *sec*, *sei*	0 (0)	1 (1.6)	0 (0)	0 (0)	0 (0)	1 (0.6)
*sea*, *seb*, *sei*, *sej*	0 (0)	1 (1.6)	0 (0)	0 (0)	0 (0)	1 (0.6)
*seb*, *sec*, *sei*, *sej*	0 (0)	2 (3.2)	0 (0)	0 (0)	0 (0)	2 (1.2)

**Table 2:** Distribution of *spa* types in *S. aureus* isolates by host animal and severity of mastitis.

*spa* Type	No. of Isolates (%)					
	Ovine (n = 97)		Caprine (n = 29)		Bovine (n = 36)	Total (n = 162)
	Clinical (n = 34)	Subclinical (n = 63)	Clinical (n = 4)	Subclinical (n = 25)	Clinical	
t012	1 (2.9)	0 (0)	0 (0)	0 (0)	0 (0)	1 (0.6)
t031	1 (2.9)	0 (0)	0 (0)	0 (0)	0 (0)	1 (0.6)
t085	0 (0)	1 (1.6)	0 (0)	0 (0)	0 (0)	1 (0.6)
t116	0 (0)	1 (1.6)	0 (0)	0 (0)	0 (0)	1 (0.6)
t1166	1 (2.9)	0 (0)	0 (0)	0 (0)	1 (2.8)	2 (1.2)
t12028	0 (0)	0 (0)	0 (0)	0 (0)	1 (2.8)	1 (0.6)
t148	0 (0)	1 (1.6)	0 (0)	0 (0)	0 (0)	1 (0.6)
t14993	0 (0)	1 (1.6)	0 (0)	0 (0)	0 (0)	1 (0.6)
t1532	0 (0)	0 (0)	0 (0)	0 (0)	1 (2.8)	1 (0.6)
t1534	0 (0)	1 (1.6)	0 (0)	1 (4.0)	1 (2.8)	3 (1.8)
t1773	7 (20.6)	4 (6.3)	1 (25.0)	0 (0)	1 (2.8)	13 (8.0)
t1896	0 (0)	2 (3.2)	0 (0)	0 (0)	1 (2.8)	3 (1.8)
t2516	0 (0)	0 (0)	0 (0)	0 (0)	1 (2.8)	1 (0.6)
t267	0 (0)	1 (1.6)	0 (0)	0 (0)	0 (0)	1 (0.6)
t2678	4 (11.8)	0 (0)	0 (0)	0 (0)	4 (11.1)	8 (4.9)
t306	1 (2.9)	2 (3.2)	0 (0)	2 (8.0)	0 (0)	5 (3.1)
t3568	1 (2.9)	6 (9.5)	0 (0)	1 (4.0)	0 (0)	8 (4.9)
t3586	8 (23.5)	18 (28.6)	0 (0)	8 (32.0)	14 (38.9)	48 (30.0)
t359	1 (2.9)	0 (0)	0 (0)	0 (0)	0 (0)	1 (0.6)
t3739	1 (2.9)	0 (0)	0 (0)	0 (0)	0 (0)	1 (0.6)
t4038	2 (5.9)	14 (22.2)	1 (25.0)	5 (20.0)	6 (16.7)	28 (17.3)
t524	3 (8.8)	0 (0)	1 (25.0)	0 (0)	0 (0)	4 (2.5)
t5428	1 (2.9)	0 (0)	0 (0)	0 (0)	0 (0)	1 (0.6)
t548	0 (0)	2 (3.2)	0 (0)	1 (4.0)	0 (0)	3 (1.8)
t5728	1 (2.9)	0 (0)	0 (0)	0 (0)	1 (2.8)	2 (1.2)
t6058	0 (0)	0 (0)	0 (0)	0 (0)	1 (2.8)	1 (0.6)
t693	0 (0)	1 (1.6)	0 (0)	0 (0)	0 (0)	1 (0.6)
t7008	1 (2.9)	1 (1.6)	0 (0)	0 (0)	0 (0)	2 (1.2)
t701	0 (0)	2 (3.2)	0 (0)	0 (0)	0 (0)	2 (1.2)
t7946	0 (0)	0 (0)	0 (0)	1 (4.0)	0 (0)	1 (0.6)
t7947	0 (0)	3 (4.8)	1 (25.0)	0 (0)	0 (0)	4 (2.5)
t891	0 (0)	1 (1.6)	0 (0)	0 (0)	0 (0)	1 (0.6)
NT	0 (0)	1 (1.6)	0 (0)	6 (24.0)	3 (8.3)	10 (6.2)

NT: not typeable due to multiple or lack of patterns of repeats.

**Table 3:** Phenotypic characteristics of *S. aureus* isolated from cases of ovine, caprine, and bovine mastitis.

Phenotype	No. of Isolates (%)					
	Ovine (n = 97)		Caprine (n = 29)		Bovine (n = 36)	Total (n = 162)
	Clinical (n = 34)	Subclinical (n = 63)	Clinical (n = 4)	Subclinical (n = 25)	Clinical	
Antimicrobial						
resistance						
Penicillin (P)	10 (29.4)	6 (9.6)	1 (25.0)	1 (4.0)	9 (25.0)	27 (16.7)
Erythromycin (E)	1 (2.9)	1 (1.6)	0 (0)	0 (0)	1 (2.8)	3 (1.9)
Tetracycline (TET)	11 (32.4)	2 (3.2)	1 (25.0)	0 (0)	9 (25.0)	23 (14.2)
Streptomycin (S)	2 (5.9)	10 (15.9)	0 (0)	3 (12.0)	2 (5.6)	17 (10.5)
Antimicrobial resistance						
patterns						
P	2 (5.9)	4 (6.3)	1 (25.0)	1 (4.0)	5 (13.9)	13 (8.0)
E	1 (2.9)	1 (1.6)	0 (0)	0 (0)	1 (2.8)	3 (4.8)
TET	5 (14.7)	0 (0)	1 (25.0)	0 (0)	5 (13.9)	11 (6.8)
S	0 (0)	8 (12.7)	0 (0)	3 (12.0)	2 (5.6)	13 (8.0)
P-S	2 (5.9)	0 (0)	0 (0)	0 (0)	0 (0)	2 (1.2)
P-TET	6 (17.6)	0 (0)	0 (0)	0 (0)	4 (11.1)	10 (6.2)
P-TET-S	0 (0)	2 (3.2)	0 (0)	0 (0)	0 (0)	2 (1.2)
Biofilm production						
ability						
No biofilm	0 (0)	0 (0)	0 (0)	0 (0)	0 (0)	0 (0)
Weak	0 (0)	6 (9.5)	0	3 (12.0)	0	9 (5.6)
Moderate	14 (41.2)	25 (39.7)	2 (50.0)	9 (36.0)	5 (13.9)	55 (34.0)
Strong	20 (58.8)	32 (50.8)	2 (50.0)	13 (52.0)	31 (86.1)	98 (60.4)
Hydrophobicity						
Low	34 (100)	63 (1000	4 (100)	25 (100)	36 (100)	162 (100)
Medium	0 (0)	0 (0)	0 (0)	0 (0)	0 (0)	0 (0)
High	0 (0)	0 (0)	0 (0)	0 (0)	0 (0)	0 (0)
Autoaggregation						
ability						
Low	13 (38.2)	55 (87.3)	0 (0)	23 (92.0)	9 (25.0)	100 (61.7)
High	21 (61.8)	8 (12.7)	4 (100)	2 (8.0)	27 (75.0)	62 (38.3)

## Data Availability

Not applicable.

## Supplementary Materials

The following are available online. Figure S1. Dendrogram of *Sma*I PFGE pulsotypes (P) and characteristics of the 162 *S. aureus* isolates, doi.org/10.5281/zenodo.4446115; Figure S2. Determination of biofilm formation capacity of staphylococcal strains, using a semi-quantitative, microtiter-plate (MTP) adherence assay, doi.org/10.5281/zenodo.4518750.
